# Sphingolipids and mitochondrial function, lessons learned from yeast

**DOI:** 10.15698/mic2014.07.156

**Published:** 2014-06-25

**Authors:** Pieter Spincemaille, Bruno P. Cammue, Karin Thevissen

**Affiliations:** 1 Centre of Microbial and Plant Genetics (CMPG), KU Leuven, Kasteelpark Arenberg 20, 3001 Heverlee, Belgium.; 2 Department of Plant Systems Biology, VIB, Technologiepark 927, 9052, Ghent, Belgium.

**Keywords:** yeast, sphingolipids, mitochondrial function, Wilson disease, Niemann Pick type C1

## Abstract

Mitochondrial dysfunction is a hallmark of several neurodegenerative diseases such as Alzheimer’s disease and Parkinson’s disease, but also of cancer, diabetes and rare diseases such as Wilson’s disease (WD) and Niemann Pick type C1 (NPC). Mitochondrial dysfunction underlying human pathologies has often been associated with an aberrant cellular sphingolipid metabolism. Sphingolipids (SLs) are important membrane constituents that also act as signaling molecules. The yeast *Saccharomyces cerevisiae *has been pivotal in unraveling mammalian SL metabolism, mainly due to the high degree of conservation of SL metabolic pathways. In this review we will first provide a brief overview of the major differences in SL metabolism between yeast and mammalian cells and the use of SL biosynthetic inhibitors to elucidate the contribution of specific parts of the SL metabolic pathway in response to for instance stress. Next, we will discuss recent findings in yeast SL research concerning a crucial signaling role for SLs in orchestrating mitochondrial function, and translate these findings to relevant disease settings such as WD and NPC. In summary, recent research shows that *S. cerevisiae *is an invaluable model to investigate SLs as signaling molecules in modulating mitochondrial function, but can also be used as a tool to further enhance our current knowledge on SLs and mitochondria in mammalian cells.

## INTRODUCTION

Aberrancies in mitochondrial function generally termed mitochondrial dysfunction are characteristic of a plethora of human pathologies such as cancer 1,2, Parkinson’s disease 3, Alzheimer’s disease 4, Friedreich’s ataxia 5, Wilson’s disease (WD) 6, metabolic syndrome and non-alcoholic fatty liver disease 7,8,9,10, diabetes 11 and drug-induced liver injury 12,13. Mitochondrial dysfunction originates from (i) inherited mutations in genes encoding subunits of the electron transport chain (ETC) located on both nuclear and mitochondrial DNA (mtDNA) 14,15, (ii) acquired mutations that arise during the normal aging process but also as a result of chronic hypoxia, viral infections, radiation, chronic stress or chemical pollution 16,17,18,19,20,21,22,23,24,25 and (iii) drug treatments such as antivirals and chemotherapeutics 12,13. Interestingly, several mitochondrial dysfunction-related conditions are associated with a perturbed sphingolipid (SL) metabolism 26,27,28,29,30,31,32,33,34. SLs are important components of cell membranes 35 and play a crucial role as signaling molecules orchestrating cell growth, differentiation and apoptosis 36,37,38.

The yeast *S. cerevisiae *(baker’s or budding yeast) has been broadly exploited as a eukaryote model organism since the publication of its genome in 1996, resulting in the annotation of approximately 6000 genes located on 16 chromosomes 39. Sequencing of the mtDNA was performed independently in 1998 40. In contrast, the human mtDNA sequence was already published in 1981 41 and in 1988 the first mtDNA mutation-related human pathology was identified as Leber’s hereditary optic neuropathy (LHON) 42. LHON is characterized by optic nerve degeneration that leads to visual impairment or blindness 43. Interestingly, approximately 31 % of the protein-coding genes in yeast have a mammalian orthologue 44 and 30 % of the genes known to be involved in human diseases may have a yeast orthologue 45,46. Remarkably, pathways that modulate apoptosis and mitochondrial function, as well as SL metabolism, are well conserved from yeast to higher eukaryotes 47,48,49,50,51,52. These aspects make yeast an extremely useful tool to study human diseases.

Given the numerous reports connecting SLs, mitochondrial (dys)function and human pathologies, and the position of *S. cerevisiae* as a model organism, we here provide an overview of literature on the interplay between SLs and mitochondrial (dys)function in the yeast *S. cerevisiae* and will translate these findings to relevant diseases characterized by mitochondrial dysfunction and/or aberrant SL metabolism. When we discuss yeast in this review, it typically refers to *S. cerevisiae.*

## MITOCHONDRIA, CELLULAR POWERHOUSES

Mitochondria are double-membraned dynamic cell organelles that constantly change shape through fusion and fission 53,54 and are present in the cytoplasm of all eukaryotic cells, except mature erythrocytes 23. The mitochondrial membranes consist of a mixture of lipids with the most abundant species phosphatidylcholine (PC), phosphatidylethanolamine (PE) and to a lesser extent cardiolipin (CL) in mammalian cells, whilst in yeast the most abundant species are PC and PE, and to a lesser extent CL and phosphatidylinositol (PI) 55. The primordial function of mitochondria is ATP production via oxidative phosphorylation (OXPHOS). However, mitochondria also play a crucial role in the regulation of cell processes such as apoptosis and cellular ion homeostasis. For more detailed descriptions on mitochondrial function and structure, the reader is referred to 56,57.

In mammalian cells, cellular energy is mainly produced via aerobic respiration, although energy can also be generated via glycolysis in absence of oxygen, which is however far less efficient 58. In contrast, tumor cells display high rates of glycolysis in the presence of sufficient oxygen, also known as the Warburg effect 59,60. Interestingly, by using specific carbon sources, yeast metabolism can either be shifted towards high glycolysis, combined glycolysis and respiration, or respiration alone by forcing growth on glucose, galactose or glycerol, respectively 61,62, which is advantageous to investigate the role of respiration in a specific cellular process.

## SPHINGOLIPIDS

In general, SLs are classified as lipids that contain a sphingoid base as the structural backbone, further decorated by a polar head group and a fatty acid side chain. Three major classes of sphingoid backbones are known: sphingosine (Sph), dihydrosphingosine or sphinganine (dhSph) and phytosphingosine (phytoSph) 63. Next to their function as membrane constituents, SLs act as important signaling molecules. Traditionally, the central SL ceramide (Cer), Cer-1-phosphate (Cer-1-P), Sph and Sph-1-phosphate (Sph-1-P) are well characterized bioactive SLs with roles in cell growth, apoptosis, inflammation, proliferation, and others 64,65,66. Intriguingly, SLs have been linked to mitochondrial function in both mammalian cells and yeast 67,68,69,70,71,72,73,74.

**Figure 1:**
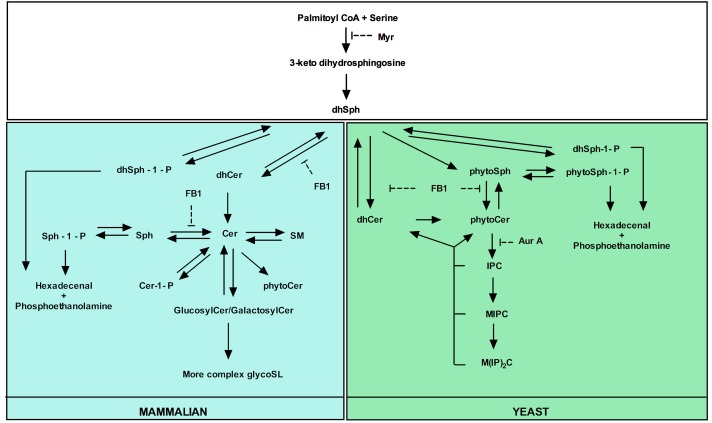
FIGURE 1: Major yeast and mammalian SL metabolic pathways. Both overlapping parts (white square) and yeast- (green square) and mammalian (blue square)-specific processes are indicated as well as the targets of commonly used inhibitors of SL biosynthesis. Adapted from 87.

Several tools have contributed to our understanding of SL metabolism, signaling and composition in mammalian and yeast cells. For instance, mass spectrometry methods are commonly used to detect different SL species and quantify their abundance in response to various stimuli 75,76. In addition, inhibitors of SL metabolism are routinely used to elucidate the role of SLs in various settings 77,78,79,80,81,82,83,84,85,86. Despite the high degree of conservation of SL metabolic pathways between mammalian and yeast cells 52,87,88, there are still yeast- and mammalian-specific aspects, and particularly in biosynthetic pathways. The major yeast and mammalian SL metabolic pathways are outlined in Fig. 1.

In the following part we subsequently describe both the mammalian and yeast SL metabolism, and discuss the use of SL biosynthetic inhibitors.

### Mammalian sphingolipid metabolism

In mammalian cells, the central SL Cer can be generated via either *de novo *biosynthesis or the salvage pathway 89 (Fig. 1). *De novo *Cer biosynthesis typically starts with the condensation of serine and palmitoyl CoA to 3-ketodihydrosphingosine by the serine palmitoyltransferase enzyme (SPT) 90. 3-Ketodihydrosphingosine is subsequently reduced to dhSph by 3-ketodihydrosphingosine reductase 91. Addition of a fatty acid side chain via an amide bond to dhSph then yields dihydroceramide (dhCer), which gets desaturated to Cer by Cer synthase 92 and dihydroceramide desaturase (dhCer desaturase) respectively 93. dhSph can also be generated from ceramidase (CDase)-mediated catabolism of dhCer 94. Cer is converted to Sph by CDase 94 or Cer-1-P by the action of Cer kinase 95. In addition, Cer serves as precursor for the formation of complex SLs such as sphingomyelin (SM) by SM synthase 96 or glucosylCer/galactosylCer by addition of phosphocholine or a carbohydrate, respectively, as polar headgroup. Glucose is incorporated by glucosylCer synthase to yield glucosylCer 97, whereas galactose is incorporated by Cer galactosyl transferase to generate galactosylCer^98^,^99^. SM interacts with cholesterol in the plasma membrane forming SM-cholesterol-rich domains and regulates cholesterol distribution in cellular membranes and cholesterol homeostasis in cells 100. GlycoSLs can function as receptor for carbohydrate binding proteins on the membrane to initiate transmembrane signaling events as well as cell growth, differentiation and cell-to-cell communication 101,102,103,104. GlucosylCer and galactosylCer can either be catabolized to Cer by glucocerebrosidase 105 or galactosylceramidase 106, respectively, or serve as precursor in the formation of more complex glycoSLs. Cer also serves as precursor in the formation of low levels phytoceramide (phytoCer) by the action of dhCer desaturase 107,108. Sphingomyelinases (SMases) break down SM to Cer 109. Sph and dhSph are phosphorylated by Sph kinases to produce Sph-1-P and dhSph-1-phosphate (dhSph-1-P), respectively 110. Cleavage of Sph-1-P and dhSph-1-P into phosphoethanolamine and hexadecenal, catalyzed by Sph-1-P lyase 111,112,113, represents the only exit route from the SL pathway. In turn, Sph1-P and dhSph-1-P are dephosphorylated by Sph-1-P phosphatase to yield Sph and dhSph, respectively 112,114, while Cer-1-P is dephosphorylated by Cer-1-P phosphatase generating Cer 115. The salvage pathway to generate Cer refers to the catabolism of complex SLs into Cer and then Sph by CDase-mediated Cer breakdown. These Sph species can be reacylated by Cer synthase to Cer 89.

### Yeast sphingolipid metabolism

*De novo *SL production is conserved from yeasts to mammals up to the synthesis of dhSph (Fig. 1) 52. In yeast, dhSph is hydroxylated to phytoSph by sphinganine C_4_-hydroxylase (Sur2p) 116. The sphingoid bases dhSph and phytosphingosine (phytoSph) are commonly referred to as long chain bases (LCBs) in yeast. Next, a fatty acid side chain is added to phytoSph or dhSph, both catalyzed by the yeast Cer synthase, i.e. Lag1p and Lac1p 117, generating phytoCer and dhCer, respectively. The former is the central SL in yeast. Sur2p-mediated hydroxylation of dhCer then generates phytoCer 116. In addition, the yeast dihydroceramidase (Ydc1p) and yeast phytoceramidase (Ypc1p) hydrolyze dhCer to dhSph 118 and phytoCer to phytoSph 119, respectively. Polar headgroups can be added to phytoCer in order to generate different species of complex SL. In yeast, three major complex SL species exist: (i) inositolphosphoceramide (IPC) is created by extending phytoCer with phospho-inositol by IPC synthase (Aur1p and Kei1p) 120, (ii) addition of mannose to IPC by mannose inositolphosphoceramide (MIPC) synthase (Csg1p , Csg2p and Csh1p) generates MIPC 121, and (iii) addition of another phospho-inositol residue to MIPC, catalyzed by inositol phosphotransferase (Ipt1), leads to the generation of mannose diinositolphosphoceramide (M(IP)_2_C) 122. Breakdown of the three complex SLs in yeast is catalyzed by inositol phosphosphingolipid phospholipase C (Isc1p) to generate phytoCer and dhCer 123,124. Next to their role as precursor in the formation of phytoCer, the LCBs dhSph and phytoSph can be phosphorylated by LCB kinases (Lcb4p and Lcb5p) to generate dhSph-1-P and phytoSph-1-P, respectively 125. The phosphorylated LCBs can then either be dephosphorylated back to dhSph and phytoSph by LCB-1-phosphate (LCB-1-P) phosphatases (Lcb3p and Ysr3p) 126,127,128, or catabolized by dhSph phosphate lyase (Dpl1) yielding phosphoethanolamine and hexadecenal 129. For a more detailed description the reader is referred to 130.

### Sphingolipid biosynthetic inhibitors

To date, the best characterized and most used inhibitors of SL biosynthesis in yeast research include Myriocin (Myr), isolated from *Myriococcum albomyces* and *Mycelia sterilia *131; Aureobadisin A (Aur A), isolated from *Aureobasidium pullulans *132; and Fumonisin B1 (FB1), isolated from *Fusarium monoliforme *133. Myr inhibits *de novo *SL biosynthesis in all eukaryotes by binding the first biosynthetic enzyme SPT 90,134,135,136, while Aur A inhibits yeast IPC synthase 137. FB1 inhibits Cer synthase in yeast and mammalian cells (Fig. 1) 138,139.

Inhibitors of specific SL biosynthetic enzymes are broadly exploited to synthetically affect different parts of the SL metabolic pathway in order to elucidate the origin of SLs in specific settings. A well-studied case has been the unraveling of the role of *de novo *SL biosynthesis during heat stress in yeast. Heat stress induces a transient cell cycle arrest in yeast 140 followed by resumption of growth at the elevated temperature 141 and several studies have implicated SLs in the heat stress response. For instance, heat stress causes a transient 2-3-fold increase of C_18_-dhSph and C_18_-phytoSph levels, an over 100-fold transient increase in C_20_-dhSph and C_20_-phytoSph, a stable 2-fold increase in C_18_-phytoSph containing Cer and a 5-fold increase in C_20_-phytoSph containing Cer 142. Dickson and coworkers observed accumulation of the disaccharide trehalose, which is essential for protection against heat stress 143. This effect is related to the LCB-induced expression of the trehalose biosynthetic gene *TPS2 *144. In addition, blocking synthesis of complex SLs by Aur A treatment, leading to an accumulation of LCBs and Cer, induces *TPS2 *expression at non-stressing temperatures. Furthermore, Aur A potentiates the effect of dhSph or heat stress on *TPS2 *expression 142. Similar findings regarding heat stress-induced accumulation of LCBs and Cer were reported by Jenkins and coworkers but also that complex SLs are unaffected while Cer levels are increased, which was partially abrogated by FB1 treatment 145. Taken together, these findings indicate a role for *de novo* SL synthesis during heat stress.

Several additional studies indicated that specific SL species fulfill different roles in the regulation of particular cellular responses. For instance, Jenkins and Hannun reported that LCBs are likely to be the active species to trigger cell cycle arrest during heat stress, which was confirmed as exogenous addition of either dhSph or phytoSph induces transient cell cycle arrest 146. In addition, during heat stress *de novo *SL biosynthesis is responsible for LCBs and phytoCer production, while Isc1p-mediated hydrolysis of complex SLs accounts for dhCer production 147. In addition, Δ*isc1 *mutants display a similar cell cycle arrest as compared to the wild type strain during heat stress, indicating that Isc1p-mediated SL generation does not affect cell cycle regulation during heat stress 147. Montefusco and coworkers addressed the specific role of distinct Cer species in SL signaling in yeast via a lipidomic and transcriptomic analysis of yeast cultures treated with different combinations of heat stress, Myr and the fatty acid myristate (C_14_) 148. Their results indicate that long chain dhCer species (C_14_ and C_16_) affect the expression of genes related to iron ion transportation while very long chain dhCer species (C_18_, C_18:1_, C_20_ and C_20:1_) are involved in the vacuolar protein catabolic process.

A role for stress-related SL signaling is not limited to heat stress in yeast, but has also been implicated in stress induced by toxic iron. Iron toxicity is directly related to the generation of deleterious reactive oxygen species (ROS) 149. Lee and coworkers linked SLs to iron toxicity as high iron increases the levels of LCBs and LCB-1-Ps, and decreasing these levels by Myr treatment increases yeast tolerance to high iron 150. These data point to a signaling role for LCBs and LCB-1-Ps in iron toxicity. In yeast, LCBs are known to directly phosphorylate protein kinases such as Pkh1p, which is redundant with Pkh2p and related to mammalian 3-phosphoinositide-dependent protein kinase PDK1 151, and Ypk1p and its paralogue Ypk2p, related to serum- and glucocorticoid-inducible kinase (SGK) 151,152. Alternatively, Ypk1/2p is phosphorylated by Pkh1p in response to LCBs. Regarding a signaling role for LCBs during iron toxicity, loss of either Pkh1p or Ypk1p indeed increases yeast tolerance to high iron 150. Hence, LCB-based SL signaling is involved in the cellular response during iron toxicity. For additional information concerning heat and iron stress in yeast and signaling pathways mediated by LCBs the reader is referred to 150,153,154,155,156. Taken together, these findings suggest that SLs fulfil a crucial signaling role during various stress conditions and that specific SL species orchestrate differential responses.

## CLUES FROM YEAST RESEARCH THAT LINK SLs TO MITOCHONDRIAL FUNCTION

The use of SL biosynthetic inhibitors in the lower eukaryotic model yeast, *S. cerevisiae*, has provided interesting insights into the interplay between SLs and mitochondrial function. For instance, Myr does not induce killing in yeast cells lacking mitochondrial DNA 157, i.e. *ρ^0 ^*cells, suggesting that decreased *de novo *SL synthesis is detrimental for cell viability and requires functional mitochondria. In addition, in yeast lifespan regulation is linked to SLs as Myr treatment extends yeast chronological lifespan (CLS), which is associated with decreased levels of LCBs, LCB-1-Ps and IPCs 78. The yeast protein kinase Sch9p is a known regulator of longevity in yeast 158 and is activated upon phosphorylation by LCBs directly or via LCB-induced activation of Pkh1/2p 151. In addition, Sch9p is phosphorylated by the action of the Target of Rapamycin Complex 1 (TORC1) 159, involved in nutrient signaling 159. The reduction in SL levels upon Myr treatment during CLS was proposed to decrease activity of the Pkh1/2p-Sch9p signaling axis resulting in an increased CLS. However, a Sch9p-independent effect on CLS was also described as Myr treatment increases CLS of Δ*sch9 *mutants 78. Subsequently, the effect of Myr on CLS was shown to be related to its effect on the yeast transcriptome. Myr treatment during yeast ageing results in the upregulation of many genes linked to mitochondrial function and oxidative phosphorylation but also to stress responses and autophagy, and downregulation of genes related to ribosomes, cytoplasmic and mitochondrial translation, as well as to ER glycoprotein and lipid biosynthesis 160. Hence targeting SL biosynthesis has provided insights in a link between SLs and regulating mitochondrial function.

Next to *S. cerevisiae, *the use of higher eukaryotic model organisms such as *Caenorhabditis elegans* has also significantly contributed to our current understanding of mammalian SL metabolism, and has pointed to a connection between SLs and mitochondrial function. Mitochondrial defects in *C. elegans *are detected by a surveillance pathway, which causes the induction of mitochondrial chaperone genes such as *hsp-*6, but also drug-detoxification genes such as *cyp-14A3* and* ugt-61 *161,162,163,164*.* As such, a RNA interference (RNAi) screen in *C. elegans* was conducted, thereby aiming at identifying genes that, upon their inactivation, renders nematodes unable to activate the mitochondrial surveillance pathway in response to mitochondrial dysfunction induced by drugs or by genetic interruption. Among their hits was *sptl-1*, encoding the *C. elegans* SPT. For instance, Sptl-1 inactivation renders nematodes unable to upregulate *hsp-6* in response to inhibition of the mitochondrial electron transport by Antimycin, while no effect on *hsp-6* is observed in absence of Antimycin 164. In addition, knockout of both Cer synthase genes decreases *hsp-6* induction upon mitochondrial damage while Myr prevents Antimycin-induced *hsp-6p* expression. Strikingly, exogenous addition of C_24_-Cer, but not dhCer or C_16_-, C_20_- or C_22_-Cer, restores the ability of *sptl-1(RNAi) *animals to trigger *hsp-6 *expression in presence of Antimycin, but not in absence of Antimycin 164. Hence, this indicates that SLs are involved in the cellular response to mitochondrial dysfunction and that distinct SLs do serve an important signaling role in modulating mitochondrial function in higher eukaryotes in general.

In mammalian cells, the specific underlying mechanisms that connect SLs, and more specifically Cer to mitochondrial function mainly remain unclear. Nevertheless, Cer species are present in mitochondria and there are various reports that link Cer species to mitochondrial function as (i) Cer species are required for ETC complex activity, but can also inhibit ETC complexes and induce the formation of reactive oxygen species (ROS), (ii) Cer species reduces the Δ*ψ_m_* by mitochondrial pore formation, triggers mitochondrial outer membrane permeabilization and thus initiates apoptosis, and (iii) Cer species are determinants for the induction of mitophagy 67. Mitophagy is a mitochondrial quality control mechanism that eliminates dysfunctional and aged mitochondria 165. Next to these aspects (i-iii) that were recently reviewed 67, other reports that link Cer species to mitochondrial function in mammalian cells include (iv) the presence of Cer-producing enzymes in the mitochondria. El Bawab and coworkers described the identification of a human CDase that localizes to the mitochondria and is ubiquitously expressed, with the highest expression levels in the kidneys, skeletal muscles and heart 166. Also, purified mitochondria and the mitochondria-associated membrane from rat liver synthesize Cer *in vitro* via Cer synthase or reverse CDase activity 167 and there are studies describing the identification of a novel SMase that displays mitochondrial localization in zebrafish and mice as discussed below 168,169. Lastly, in addition to the above-mentioned links between Cer and mitochondrial function (i-iv) there are (v) reports that link Cer species to mitochondrial fission events. Mitochondrial fusion is a compensatory mechanism to decrease stress by mixing the contents of partially damaged mitochondria, while mitochondrial fission is referred to as mitochondrial division in order to create new mitochondria. Both mitochondrial fusion and fission are closely involved in cell processes such as mitophagy, cell death and respiration 170. As described by Parra and coworkers, in contrast to C_2_-dhCer, C_2_-Cer induces rapid fragmentation of the mitochondrial network in rat cardiomyocytes and increased mitochondrial content of the mitochondrial fission effectors Drp1 and Fis1 171,172. Additionally, inhibition of Cer synthase decreases recruitment of Drp1 and Fis1 to the mitochondria and concomitantly also reduces mitochondrial fission 173. Moreover, Smith and coworkers showed that C_2_-Cer addition causes rapid and dramatic division of skeletal muscle mitochondria, which is characterized by increased Drp1 expression and reduced mitochondrial respiration. Interestingly, these effects are abrogated by Drp1 inhibition 174. These reports directly link Cer species to mitochondrial fission. Taken together, there is abundant evidence that links SLs to mitochondrial function in mammalian cells.

In the following part we will first describe novel findings with regard to the SL-mitochondria connection using yeast as a model and translation of these findings to relevant higher eukaryotic settings related to mitochondrial (dys)function. We will hereby focus on Isc1p and Ncr1p, the yeast orthologue of the Niemann Pick type C1 (NPC) disease protein 175. Also, in the context of WD, a pathological condition characterized by excess Cu and mitochondrial dysfunction 176, we will describe the potential of yeast as a model to identify novel compounds that can inhibit Cu-induced apoptosis in yeast.

## Inositol phosphosphingolipid phospholipase C (Isc1p) and mitochondrial function in *S. cerevisiae *

In *S. cerevisiae, *several reports have linked SLs to mitochondrial function via the action of Isc1p 68,69,70,71,72,73. Reportedly, Isc1p mainly resides in the ER, but localizes to the outer mitochondrial membrane during the late exponential and post-diauxic growth phase 68,71,177,178. Isc1p is homologous to the mammalian neutral SMases (nSMase) 124. Interestingly, a novel SMase in zebrafish cells was identified that localizes to the intermembrane space and/or the inner mitochondrial membrane 168. Furthermore, a novel murine nSMase was reported to localize to both mitochondria and ER termed mitochondria-associated nSMase (MA-nSMase) 169. MA-nSMase expression varies among tissues and like Isc1p in yeast 123,179 its activity is highly influenced by phosphatidylserine and CL 169. Though a human MA-nSMase has not yet been characterized, a putative human MA-nSMase encoding gene has been identified 180. It is therefore conceivable that the MA-nSMase is the mammalian counterpart of yeast Isc1p, though this has yet to be elucidated as well as a putative role for human MA-nSMase in regulating mitochondrial function by modulating SL-levels.

Isc1p and its associated SL species have been extensively studied as regulators of mitochondrial function as several studies demonstrated that Δ*isc1 *mutants display several markers of mitochondrial dysfunction such as a decreased CLS 70, the inability to grow on a non-fermentable carbon source 69,71,72,181,182, increased frequency of petite formation 178, mitochondrial fragmentation 72 and abnormal mitochondrial morphology 73. Furthermore, Δ*isc1 *mutants display an aberrant cellular and mitochondrial SL composition as Δ*isc1 *mutants exhibit decreased levels of all SLs with the most striking decreases in α-OH-C_24_-phytoCer and α-OH-C_26_-phytoCer species, while α-OH-C_14_-phytoCer and C_26_-phytoCer levels are increased 178. In addition, Δ*isc1 *mutants are characterized by decreased dhSph and α-OH-phytoCer levels and increased C_26_-dhCer and C_26_-phytoCer levels during CLS 181. Strikingly, exogenous addition of C_12_-phytoCer allows Δ*isc1 *mutants to grow on a non-fermentable carbon source 71. In line with Cowart and coworkers who reported that Δ*isc1 *mutants display aberrant gene regulation 147, Kitagaki and coworkers revealed that mitochondrial dysfunction related to loss of Isc1p is caused by a misregulation of gene expression rather than an inherent mitochondrial defect as Δ*isc1 *mutants are unable to up-regulate genes that are involved in non-fermentable carbon source utilization, and down-regulate genes related to nutrient uptake and amino acid metabolism 182. This points to an important signaling role for Isc1p-mediated SL generation in regulating mitochondrial function in yeast.

Currently identified downstream signaling proteins related to perturbed mitochondrial function in Δ*isc1 *mutants include the type 2A-related serine-threonine phosphatase Sit4p 183, the mitogen-activated protein kinase Hog1p, involved in response to hyperosmotic stress 184,185,186,187, and the TORC1/Sch9p pathway 69,72,177,181. The TORC1/Sch9p signaling pathway however is proposed as the central signaling axis to pass upstream SL signals to downstream effectors such as Hog1p and Sit4p that affect mitochondrial function 72. It is likely that additional signaling pathways are also involved in modulating mitochondrial function in response to SLs, however, these pathways have yet to be identified. For more information concerning our current knowledge on how SLs related to the action of Isc1p are implicated in regulating mitochondrial function, and the role of the aforementioned signaling proteins the reader is referred to 74.

## *S. cerevisiae *Δ*ncr1* mutants, a model for Niemann Pick type C1

NPC is a fatal lipid storage disease with progressive neurodegeneration that affects 1/150.000 live births 188. While neurodegeneration is the most prominent feature of NPC, organs such as the liver, ovaries and lungs also display aberrant lipid storage 189. NPC is typically caused by mutations in the genes encoding NPC1 and NPC2 accounting for 95 % and 5 % of all cases, respectively 190,191,192. NPC1 and NPC2 remove cholesterol from the late endosomes/lysosomes (LE/LY) 191,192. Cholesterol is a sterol involved in membrane function modulation and precursor to steroid hormones, oxysterols and vitamin D 193. NPC1-deficient cells tend to accumulate lipids such as cholesterol, glycoSL and Sph in the LE/LY 79,194,195. Despite the facts that the specific mechanisms leading to neurodegeneration in NPC are not well established, mitochondrial dysfunction and oxidative stress are found to be key characteristics of NPC 196,197,198,199,200. Intriguingly, a pharmacological approach targeting glycoSL synthesis alleviates symptoms in NPC animal models 201, however, an underlying effect on mitochondrial function was not addressed. Hence, targeting SL homeostasis might be a promising approach in treatment of NPC.

Given the conservation of NPC1 and NPC2 in eukaryotes, several non-mammalian models are available to study NPC including the model yeast *S. cerevisiae* 202. In yeast, Ncr1p (NPC1 related gene 1) is the orthologue of NPC1 175 and localizes to the membrane of the vacuole 203. The role of Ncr1p has been described as fundamentally linked to SL homeostasis with sterol movement as a consequence 175,202. Yeast does not synthesize cholesterol, but the structural relative ergosterol 204. Whether or not the loss of Ncr1p in yeast causes ergosterol accumulation has to be clarified yet, as Malathi and coworkers showed that Δ*ncr1* mutants do not exhibit aberrancies in sterol metabolism 175 while more recently two independent research groups showed the contrary 205,206. Still, intracellular sterol transport has been linked to mitochondrial function in yeast 207. In contrast to Δ*ncr1* mutants, mutations in the putative sterol-sensing domain of Ncr1p causes several phenotypes such as impaired growth at elevated temperatures, increased salt sensitivity and low growth on acetate and ethanol as carbon source 175. Such phenotypes were ascribed to alterations in SL metabolism 175, as observed in NPC 79. Although initial studies with Δ*ncr1* mutants did not show any observable phenotype specifically related to loss of Ncr1p but rather associated with Ncr1p mutations, Berg and coworkers reported that Δ*ncr1* mutants are resistant to the ether lipid drug edelfosine 208.

Nevertheless, Vilaça and coworkers reported very recently on phenotypes of Δ*ncr1* mutants that at least partly resemble cellular alterations/aspects observed in NPC patients. For instance, Δ*ncr1* mutants display increased hydrogen peroxide sensitivity and shortened CLS, with increased prevalence of oxidative stress markers 205. Also, their results indicate that Δ*ncr1* mutants display mitochondrial dysfunction as these mutant cells are for instance unable to grow on a non-fermentative carbon source, display decreased Δ*ψ_m_* and mitochondrial fragmentation 205. In addition, Δ*ncr1* mutants display aberrant SL homeostasis as such mutants accumulate LCBs due to increased turnover of complex SLs 205. Taken together, in line with NPC 79,196,197,198,199,200, Δ*ncr1 *mutants display markers of oxidative stress, mitochondrial dysfunction and accumulate SLs.

Mitochondrial function in Δ*ncr1* mutants is suggested to be regulated by SLs. Characteristic for Δ*ncr1* mutants is the increased Pkh1p-dependent activation of Sch9p. Concomitantly, Δ*ncr1*Δ*pkh1 *and Δ*ncr1*Δ*sch9 *mutants display restored mitochondrial function as these double mutants are for instance able to grow on a non-fermentable carbon source 205. Thus, as suggested for Δ*isc1 *mutants 74, this indicates that Sch9p is involved in regulating mitochondrial function in response to SLs in Δ*ncr1 *mutants. Taken together, these results suggest that SLs indeed are essential determinants of mitochondrial dysfunction associated with NPC.

Next to the above described study, yeast studies have shed light on new potential targets for treatment of NPC. Munkacsi and coworkers identified 12 pathways and 13 genes that are of importance for growth of Δ*ncr1* mutants during anaerobis in presence of exogenous ergosterol 209. *S. cerevisiae *cells become auxotrophic to sterol in absence of oxygen 210. Based on their results, they hypothesized that histone deacetylation contributes to the pathogenesis of NPC and indeed confirmed this in NPC-derived fibroblasts: genes encoding histone deacetylases (HDACs) are upregulated in NPC-derived fibroblasts. Histone deacetylase (HDAC) plays a key role in gene regulation by removing acetyl groups from specific lysine residues on histones, which increases DNA condensation and thus thereby decreases gene expression. The opposite reaction is catalyzed by histone acetyltransferases and this increases gene expression 211,212. Intriguingly, Sph kinase 2 (SphK2), one of the two Sph kinase isoforms which mainly localizes to the nucleus 213, has been shown to associate with HDAC1 and HDAC2, two class I HDACs 214, in repressor complexes as well as histone H3 and thereby increasing H3 acetylation and transcription. This increase in H3 acetylation and transcription is attributed to the SphK2-dependent Sph-1-P production in the nucleus which directly binds to the active site of HDAC1 and HDAC2, and thereby inhibits their activity and linking SLs to gene expression 215. In addition, Munkacsi and coworkers could reverse aberrant HDAC function and show concomitant improved NPC characteristics such as decreased accumulation of cholesterol and SLs 209. In line, HDAC inhibitors were recently suggested as a promising therapeutic in treatment of NPC 216. Hence, the study by Munckacsi and coworkers indicates that *S. cerevisiae* is a powerful tool to identify novel pathways involved in the pathogenesis of NPC and for selecting novel therapeutic targets and therapies.

## *S. cerevisiae* as a model to study Cu toxicity in context of Wilson disease

WD is a relevant human pathology (incidence 1/30.000) caused by mutations in the gene encoding the Cu-transporting ATPase ATP7B resulting in the accumulation of excess Cu in the liver and increased intracellular Cu levels 176,217,218,219. This results in acute liver failure or cirrhosis but also neurodegeneration 217,218,220. Interestingly, the yeast *CCC2* gene, encoding a P-type Cu-transporting ATPase, is homologous to *ATP7B *221. Cu uptake in yeast is mediated by the high-affinity Cu transporter Ctr1p 222 and Cu is subsequently delivered to Ccc2p by the action of the Cu metallochaperone Atx1p 223. Ccc2p transports Cu to the Golgi lumen for Cu incorporation into Fet3p, which is required for iron uptake 224. Loss of Ccc2p results in respiration defects and defective iron uptake 224,225. Also, Δ*ccc2 *mutants exhibit defective growth on low iron-containing growth media which can be rescued by overexpression of wild type *ATP7B* or WD-related *ATP7B* mutants 226,227. However, *ATP7B* mutants do not restore Δ*ccc2 *mutant growth on low iron-containing growth medium to the same extent as wild type *ATPB* 226,227. Mechanistic events that are characteristic for Cu-induced toxicity in liver cells is Cu-induced mitochondrial dysfunction 6 and Cu-induced increased acid SMase (aSMase) activity 30. The latter study showed that Cu increases aSMase acitivity resulting in increased levels of pro-apoptotic Cer 30,228. In addition, their results show that aSMase inhibition, either by pharmacological intervention or genetic disruption prevents Cu-induced apoptosis 30. Interestingly, there is an increased constitutive activation of aSMase in plasma of WD patients. Thus, Cu-induced toxicity is fundamentally linked to mitochondrial dysfunction and aberrant SL metabolism.

We recently showed that the *A. thaliana*-derived decapeptide OSIP108 229 prevents Cu-induced apoptosis and oxidative stress in yeast and human cells 230, but also prevents Cu-induced hepatotoxicity in a zebrafish larvae model (unpublished data). Based on the observation that OSIP108 pretreatment of HepG2 cells was necessary in order to observe anti-apoptotic effects, we investigated the effect of OSIP108 on SL homeostasis in HepG2 cells and found that OSIP108-treated HepG2 cells displayed decreased levels of sphingoid bases (Sph, Sph-1-P and dhSph-1-P), dhCer species (C_12_ and C_14_), Cer species (C_18:1_ and C_26_) and SM species (C_14_, C_18_, C_20:1_ and C_24_). Of note is that dhSph levels in OSIP108-treated HepG2 cells were also decreased but not to a significant level. These observations led to the hypothesis that OSIP108 might act as a 3-ketodihydrosphingosine reductase inhibitor. Hence, we subsequently validated these observations in *S. cerevisiae* and found that exogenous dhSph addition abolished the protective effect of OSIP108 on Cu-induced toxicity in yeast cells 230. As exogenous dhSph abolished this protective effect, this suggests that SLs are directly involved in Cu-induced toxicity in yeast and mammalian cells, and that compounds that can rescue Cu-induced toxicity in yeast seem to specifically target SL homeostasis. There is however not yet conclusive evidence to support this hypothesis.

In addition, our ongoing research is aimed at identifying novel compounds that increase yeast tolerance to suggested inducers of mitochondrial dysfunction, including Cu. As such, by screening the Pharmakon 1600 repositioning library, we identified at least 1 class of off-patent drugs that prevent Cu-induced toxicity in yeast (unpublished data). Thus far, this drug class has not been linked to Cu toxicity, nor does their mammalian target have a yeast counterpart. We are currently translating these data to a higher eukaryotic setting. Hence, this indicates that our Cu-toxicity yeast screen can result in the identification of new novel therapeutic options and unknown targets in treatment of, for instance, WD.

## CONCLUSION

In conclusion, several studies in *S. cerevisiae *indicate an important signaling role for SLs in maintaining correct mitochondrial function. These data were confirmed in relevant mammalian models for pathologies characterized by mitochondrial dysfunction. More specifically, knowledge on the link between SLs and mitochondrial function generated in the model yeast *S. cerevisiae* advanced research in particular diseases such as WD and NPC. In addition, using yeast as screening model for these diseases, development of novel therapies seems feasible and promising.

Noteworthy is, however, that different SL species clearly have different roles as exemplified by the differential effect of Cer species with different chain length on the induction of the mitochondrial surveillance pathway in *C. elegans* 164. Moreover*, *the differential role of Cer species with different chain length in human diseases was discussed recently 231. As for yeast research, the study by Montefusco and coworkers showed that specific groups of Cer species that vary in side chain and hydroxylation coordinate different sets of functionally related genes 148. Thus, besides the fact that different Cer species are subjected to regulation by specific biochemical pathways in specific subcellular compartments, they also serve distinct roles, which was discussed previously by Hannun and Obeid 89. In the latter review article, the interconnectivity of the SL metabolism was also highlighted, given the fact that manipulating one enzyme involved in SL metabolism not only leads to the perturbation of its derived SL metabolite, but also to downstream derived SL species, denoted as the ‘metabolic ripple effect’. Hence, despite our extensive knowledge on SL metabolism and functioning, the concept of many ceramides and the interconnectivity of SL metabolism introduces additional complexity in tackling the roles for specific SL species in SL signaling.

In conclusion, basic yeast research has provided important clues for SL signaling events that impact on mitochondrial function, in higher eukaryotic and mammalian cells, as well as for novel therapeutic options for diseases in which mitochondrial dysfunction is critical.
